# Phylogenetic conservation of soil bacterial responses to simulated global changes

**DOI:** 10.1098/rstb.2019.0242

**Published:** 2020-03-23

**Authors:** Kazuo Isobe, Nicholas J. Bouskill, Eoin L. Brodie, Erika A. Sudderth, Jennifer B. H. Martiny

**Affiliations:** 1 Graduate School of Agricultural and Life Sciences, The University of Tokyo, Tokyo, Japan; 2 Climate and Ecosystem Sciences Division, Lawrence Berkeley National Laboratory, Berkeley, CA, USA; 3 Department of Environmental Science, Policy and Management, University of California, Berkeley, CA, USA; 4 Center for Environmental Studies, Brown University, Providence, RI, USA; 5 Department of Ecology and Evolutionary Biology, University of California, Irvine, CA, USA

**Keywords:** soil microbiome, global change, field experiments, phylogeny

## Abstract

Soil bacterial communities are altered by anthropogenic drivers such as climate change-related warming and fertilization. However, we lack a predictive understanding of how bacterial communities respond to such global changes. Here, we tested whether phylogenetic information might be more predictive of the response of bacterial taxa to some forms of global change than others. We analysed the composition of soil bacterial communities from perturbation experiments that simulated warming, drought, elevated CO_2_ concentration and phosphorus (P) addition. Bacterial responses were phylogenetically conserved to all perturbations. The phylogenetic depth of these responses varied minimally among the types of perturbations and was similar when merging data across locations, implying that the context of particular locations did not affect the phylogenetic pattern of response. We further identified taxonomic groups that responded consistently to each type of perturbation. These patterns revealed that, at the level of family and above, most groups responded consistently to only one or two types of perturbations, suggesting that traits with different patterns of phylogenetic conservation underlie the responses to different perturbations. We conclude that a phylogenetic approach may be useful in predicting how soil bacterial communities respond to a variety of global changes.

This article is part of the theme issue ‘Conceptual challenges in microbial community ecology’.

## 1. Introduction

Soil bacterial communities play critical roles in ecosystem functioning such as carbon transformation and stabilization, nutrient and biogeochemical cycling and plant host defence. The composition of these communities is sensitive to a variety of global changes, and such shifts can alter their functioning [1,2]. A predictive understanding of how these communities respond to their environment is therefore of great interest. Among a variety of obstacles, the enormous diversity of soil bacteria creates a challenge for making predictions.

**Figure 1.**
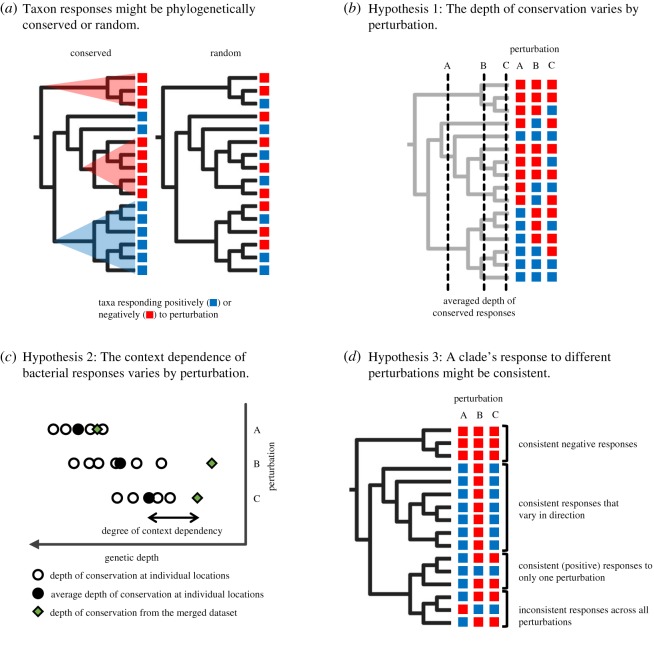
Conceptual framework for the study's three hypotheses. Bacterial taxa respond either positively (blue) or negatively (red) in their relative abundance to a perturbation. (*a*) The responses to a perturbation might be phylogenetically conserved (left) or random (right). (*b*) Hypothesis 1: The depth of conservation of the responses (the average phylogenetic depth of clades responding positively or negatively) varies by type of perturbation. Here, the response to perturbation A is more deeply conserved than the response to perturbations B or C. (*c*) Hypothesis 2: The degree to which a bacterial response is context dependent might depend on the type of perturbation. The degree of context dependency is assessed by comparing the difference between the average depth of responses at individual locations (solid circles) and the depth of responses when the datasets are merged across locations (filled green diamonds). In this hypothetical example, the context dependence of perturbation A is smaller than that of perturbations B and C. (*d*) Hypothesis 3: The consistency of a clade's response to different perturbations. Clades might respond consistently (responding generally positively or negatively) to multiple perturbations, consistently to just one perturbation, or inconsistently to all.

Phylogenetic information might simplify this diversity by offering a structure to its underlying biological variation or traits [3]. In particular, if large phylogenetic clades of bacterial taxa (such as amplicon sequence variants (ASVs) or 97% operational taxonomic units (OTUs)) respond in a similar manner, then one could reduce the number of bacterial groups considered. In such a case, the response would be said to be *phylogenetically* conserved, defined here as positive or negative responses that are non-randomly distributed across the bacterial phylogenic tree [4]. The more deeply conserved the response (the greater genetic depth at which descendant bacterial taxa show a similar response), the fewer groups that one would need to track as they could be lumped into broader taxa (figure 1*a*).

More generally, such a phylogenetic signal demonstrates a tendency for more closely related taxa to be more similar in their traits than less closely related taxa [5]. Indeed, bacterial traits are consistently phylogenetically patterned [3,6,7], despite that horizontal gene transfer and rapid evolution may break up any pattern [8,9]. Specifically, several recent studies have found that bacterial responses to environmental changes are also phylogenetically conserved [10–13] such that closely related taxa respond more similarly to a perturbation than those that are distantly related. We recently conducted an analysis of soil bacterial response to experimental N addition at 13 locations across five continents [14]. The bacterial response to added N at individual locations was phylogenetically conserved across the tree of life; closely related bacterial taxa on the tree responded more similarly to N addition than distantly related taxa. Further, we found that the phylogenetic pattern of responses was *context independent* [14]. Phylogenetic clades generally responded in the same direction (positively or negatively) in different locations, even as the baseline environment or surrounding microbial community varied. As a result, we could identify the taxonomy of larger phylogenetic clades that responded to N addition in the same direction in a variety of experimental locations. Yet increased N availability is just one way that soil ecosystems may change in the future [15–17]. We therefore wanted to expand on this prior work to consider whether a phylogenetic approach might be more predictive of bacterial responses for some global changes than others.

To do this, here we re-analysed publicly available data on soil bacterial community composition from field experiments that simulated a variety of global changes, including warming, drought, elevated atmospheric CO_2_ concentration, P addition and increased soil pH. This wider collection of types of experiments allowed us to test three new hypotheses. First, we hypothesized that the responses to each type of perturbation would vary in the depth at which they are conserved (figure 1*b*). While a response is not a trait itself, we reasoned that the degree of conservation is based on the bacterial traits underlying the response, which do vary [3]. For instance, previous studies suggest that N uptake rate is more deeply conserved than the preference for soil temperature [3,18,19]. However, a systematic comparison of the phylogenetic depth of responses across many perturbation types has not been done.

Second, we hypothesized that the degree to which a bacterial response is context dependent depends on the type of perturbation (figure 1*c*). We tested this hypothesis by comparing the average depth of responses at individual locations to the depth of responses when the datasets for each treatment type were merged (as in [14]). If the bacterial responses were highly context dependent, then the averaged phylogenetic depth in the merged dataset would be shallower than the individual locations. We reasoned that the importance of the surrounding context might depend on the degree to which a perturbation directly impacted bacterial physiology and growth. For instance, increased drought might directly select for bacteria with drought-adaptation traits such as the ability to accumulate osmolytes, produce exopolysaccharides or form thick cell walls [20–23]. Similarly, P addition might directly select for bacteria with traits for enhanced P acquisition [24,25]. By contrast, the primary effects of elevated CO_2_ and warming on soil bacteria might be mediated through the response of the surrounding plant community through changes in litter or root exudate chemistry [26,27]. Thus, the bacterial traits selected for by the perturbation may depend on the baseline nutrient environment at each location. In addition, we expected that variation in the experimental manipulations across studies might increase the context dependency of response. For instance, the warming experiments increased temperature between 1.5 and 5°C for anywhere between 1 year and 20 years; by contrast, the P addition experiments all received the same amount and type of P for 2–4 years.

Finally, we hypothesized that, while a clade's response to different perturbations would not generally be correlated [28,29], some clades might be consistently sensitive to environmental changes and therefore several types of perturbations (figure 1*d*). This pattern might occur if a clade is adapted to responding positively to environment change––for instance, possessing traits that allow a bacterium to turn on reproduction quickly, persist under stressful conditions or use a wide range of substrates [20,30]. Alternatively, a clade might respond consistently negatively if members are highly specialized to their preferred environment, such that any change in their conditions results in a decline in abundance.

## 2. Material and Methods

### (a) Study inclusion criteria

We searched for published studies that assessed soil bacterial community composition within global change field experiments. The experiments manipulated the soil environment by warming, rain exclusion (drought), elevating atmospheric CO_2_ concentration, P addition and liming (increasing pH). Each study met the following criteria: (i) published before 1 June 2019, (ii) included at least three replicates for manipulation and control (non-addition) plots, (iii) used high-throughput amplicon sequencing containing the V4, V3–V4 or V4–V5 region of 16S rRNA genes in bacteria, and (iv) sampled from surface soil (within top 15 cm). We identified 27 published studies but excluded 14 because the raw sequence datasets and/or accompanying metadata were not publicly deposited or otherwise obtainable ((table 1; electronic supplementary material, table S1). Ultimately, we included data from six locations for warming, seven locations for drought, six locations for elevating atmospheric CO_2_ concentration and six locations for P addition (table 1; electronic supplementary material, figure S1). Although we had only one location for liming, we included the data because soil acidification is also a global problem [42] and bacterial pH preference is thought to be a particularly deeply conserved trait [3]. The specific samples used at each location are summarized in electronic supplementary material, table S1.

**Table 1.** Characteristics of study locations and number of replicate plots and OTUs used in this study (control, treatment).

perturbation	location	habitat (country)	treatment	duration	no. replicate plots^a^	no. OTUs
warming	Che [31]	grassland (China)	plus 1.57°C	6 years	4, 4	2046
	DeAngelis_1 [32]	temperate forest (USA)	plus 5°C	5 years	4, 4	1138
	DeAngelis_2 [32]	temperate forest (USA)	plus 5°C	8 years	4, 4	1062
	DeAngelis_3 [32]	temperate forest (USA)	plus 5°C	20 years	4, 4	1046
	Waghmode [33]	cropland (China)	plus 1.5°C	7 years	6, 6	2298
	Zhang [29]	grassland (China)	plus 2°C	1 year	6, 6	1372
drought	Bastida_1 [34]	forest (Spain)	rainfall exclusion	6 years	6, 6	1631
	Bastida_2 [34]	forest (Spain)	rainfall exclusion	6 years	6, 6	1698
	Bouskill_1 [35]	forest (Puerto Rico)	rainfall exclusion	4 years	3, 3	934
	Bouskill_2	desert (USA)	rainfall exclusion	3–10 months	10, 20	1730
	Fernandes_1 [36]	desert soil crust (USA)	rainfall exclusion	3 years	10, 10	1903
	Fernandes_2 [36]	desert soil crust (USA)	rainfall exclusion	3 years	10, 10	2453
	Zhang [29]	grassland (China)	rainfall exclusion	1 year	6, 6	1612
elevated CO_2_	Deng [37]	grassland (USA)	368 ppm, 560 ppm^a^	10 years	12, 12	1058
	Raut_1 [38]	grassland (USA)	380–250 ppm, 500–380 ppm^a^	9 years	11, 16	1082
	Raut_2 [38]	grassland (USA)	380–250 ppm, 500–380 ppm^a^	9 years	18, 14	1051
	Raut_3 [38]	grassland (USA)	380–250 ppm, 500–380 ppm^a^	9 years	12, 12	956
	Xia [39]	grassland (New Zealand)	ambient, 475 ppm^a^	12 years	3, 3	708
	Yang [40]	grassland (USA)	ambient, ambient + 275 ppm^a^	4 years	4, 4	1447
P addition	Leff_1 [24]	grassland (Switzerland)	100 kg P ha^−1^ yr^−1^ as Ca(H_2_PO_4_)_2_	3 years	6, 3	2069
	Leff_2 [24]	grassland (Australia)	100 kg P ha^−1^ yr^−1^ as Ca(H_2_PO_4_)_2_	3 years	6, 3	1307
	Leff_3 [24]	grassland (South Africa)	100 kg P ha^−1^ yr^−1^ as Ca(H_2_PO_4_)_2_	3 years	6, 3	1232
	Leff_4 [24]	grassland (South Africa)	100 kg P ha^−1^ yr^−1^ as Ca(H_2_PO_4_)_2_	2 years	3, 3	1530
	Leff_5 [24]	grassland (South Africa)	100 kg P ha^−1^ yr^−1^ as Ca(H_2_PO_4_)_2_	2 years	6, 3	1545
	Leff_6 [24]	grassland (Australia)	100 kg P ha^−1^ yr^−1^ as Ca(H_2_PO_4_)_2_	4 years	6, 3	1275
liming	Guo [41]	paddy (China)	7500 kg ha^−1^ of CaCO_3_	>1 year	4, 4	945

^a^control plots, treatment plots.

### (b) Sequence processing

Sequence data (FASTQ-formatted raw sequence or FASTA-formatted denoised sequence files) and associated metadata were either shared by the original authors or downloaded from public databases. All of the datasets were sequenced on the Illumina MiSeq or Roche 454 platforms. In general, results from these platforms are comparable [43], and the sequencing error rates of both platforms are low relative to sequence differences between our OTU classifications (see below).

To allow comparisons across studies, we reanalysed the sequence data in a consistent manner. We trimmed all sequences to the V4 region of 16S rRNA genes that corresponds to the region amplified with 515F (GTGYCAGCMGCCGCGGTAA)/806R (GGACTACNVGGGTWTCTAAT) primers, after removing the primers and sequences outside of the target region with the Cutadapt toolkit [44]. The UPARSE pipeline [45] was used to merge the paired-end sequences of FASTQ-formatted raw sequence files, conduct quality filtering and cluster the sequences into OTUs. A minimum overlap of 20 bp was set for merging the sequences. A maximum per sequence expected error frequency value of 1.0 was set for quality filtering the sequences. Singleton sequences were removed. Paired-end sequences from all locations within a perturbation experiment (e.g. for all warming studies or for all drought studies) were merged and clustered into OTUs at ≥ 97% sequence similarity, and chimeric sequences were removed at the step of OTU clustering. Taxonomy of the representative sequence of each OTU was assigned within QIIME using the RDP classifier [46] at 80% confidence threshold trained on the latest version of SILVA database (v. 132, https://www.arb-silva.de/download/archive/qiime/). OTUs assigned as chloroplasts or mitochondria, unassigned at kingdom level, and Archaea were removed. The archaeal OTUs were relatively rare and archaeal 16S rRNA genes are known to be preferentially amplified among the primer pairs used [47].

Using a 97% OTU definition (rather than ASVs as in [48]) allowed us to compare the same taxon across many locations, which was key to our analysis. However, we also tested the sensitivity of our results by reanalysing the warming experiments using ASVs with the DADA2 pipeline (v. 1.12) [48]. The values of *τ*_D,_ the mean phylogenetic depth of clades across a tree of life sharing either positive or negative responses to a particular perturbation (figure 1*b* and see below), were 0.5–0.6 times lower when using the ASV pipeline; this result is to be expected because the abundance of each ASV is generally lower than each OTU and the response ratios of low abundance taxa would be subject to a high degree of noise. However, the *τ*_D_ values based on 97% OTUs and ASVs for each experimental location were strongly positively correlated for both positive (*R*^2^ = 0.88) and negative (*R*^2^ = 0.83) responses. This tight correlation suggests that our conclusions are robust to the OTU definition used [2].

### (c) Overall community composition responses at each location

We first tested whether the perturbations altered overall community composition at each location. Because we obtained a variable number of sequence reads per sample within each location, the sequence data were rarefied to the lowest number of reads per location to account for the variation of samples within each location (table 2). The Bray–Curtis dissimilarity metric was used to calculate compositional differences between each sample from the rarefied OTU tables with the vegan package [49] in the R environment (v. 3.6.0, http://www.R-project.org). We tested for differences in bacterial community composition between manipulation and control plots with a permutational multivariate analysis of variance (PERMANOVA) test with 999 permutations using the vegan package. Note that our PERMANOVA results could differ from the original study; such discrepancies could arise because we sometimes used only a subset of samples based on the treatment of interest, as well as differences due to sequence processing.

**Table 2.** Permutational multivariate analysis of variance (PERMANOVA) and consenTRAIT results comparing control and treatment plots by each study location. Bold values indicate a significant response (*p* < 0.05); bold italicized values indicate a highly significant response (*p* < 0.005). The consenTRAIT statistic (*τ*_D_) is given for both positive and negative responding consensus clades, defined as clades in which >90% of the descendant OTUs show the same direction of response.

perturbation	location	PERMANOVA		consenTRAIT	
		no. of rarefied sequences	*R*^2^	*τ*_D_ of positive response	*τ*_D_ of negative response
warming	Che [31]	14 577	***0***.***131***	***0***.***020***	***0***.***021***
	DeAngelis_1 [32]	9370	0.286	***0***.***020***	***0***.***017***
	DeAngelis_2 [32]	6226	0.206	0.018	**0**.**017**
	DeAngelis_3 [32]	8285	0.165	0.017	***0***.***019***
	Waghmode [33]	13 408	***0***.***218***	***0***.***022***	***0***.***021***
	Zhang [29]	2275	0.100	**0**.**022**	0.020
	merging locations	—	—	**0**.**019**	**0**.**018**
drought	Bastida_1 [34]	9702	***0***.***195***	**0**.**019**	***0***.***020***
	Bastida_2 [34]	9647	**0**.**130**	**0**.**019**	0.020
	Bouskill_1 [35]	1225	0.168	***0***.***019***	0.018
	Bouskill_2	5933	0.045	0.017	***0***.***020***
	Fernandes_1 [36]	35 695	***0***.***335***	***0***.***018***	***0***.***019***
	Fernandes_2 [36]	38 303	**0**.**111**	***0***.***018***	***0***.***021***
	Zhang [29]	2228	0.118	0.023	0.022
	merging locations	—	—	**0**.**017**	**0**.**019**
elevated CO_2_	Deng [37]	211	0.043	0.018	***0***.***021***
	Raut_1 [38]	2729	**0**.**058**	**0**.**019**	***0***.***022***
	Raut_2 [38]	2168	***0***.***067***	***0***.***021***	***0***.***022***
	Raut_3 [38]	2065	***0***.***098***	***0***.***022***	0.019
	Xia [39]	2054	0.202	0.020	0.018
	Yang [40]	5790	0.179	0.018	***0***.***019***
	merging locations	—	—	0.019	**0**.**019**
P addition	Leff_1 [24]	27 667	0.123	0.019	0.019
	Leff_2 [24]	16 881	**0**.**191**	**0**.**019**	**0**.**019**
	Leff_3 [24]	17 984	0.150	**0**.**020**	0.018
	Leff_4 [24]	18 855	0.146	0.018	0.017
	Leff_5 [24]	15 168	**0**.**191**	0.017	***0***.***018***
	Leff_6 [24]	18 855	0.069	0.017	0.020
	merging locations	—	—	0.016	***0***.***019***
liming	Guo [41]	3230	0.177	***0***.***024***	**0**.**020**

### (d) Individual operational taxonomic unit responses at each location

To quantify the response of bacterial OTUs to a perturbation at each location, we used the full sequence dataset (not rarefied) and the DESeq2 package [50] in the platform of the phyloseq package [51] in the R environment. We used DESeq2 in a limited way that differs from its typical use for RNA-Seq data. Specifically, we used it to (i) normalize the sequence counts by sample within a location by replacing the original counts with variance stabilized counts and (ii) calculate the log_2_-fold ratio of averaged relative abundance in manipulation plots relative to control plots for each taxon. Before calculating the response ratios, we first removed rare OTUs present in less than half of all the plots within a location (electronic supplementary material, table S1), as the response ratios of low occupancy, low abundance taxa would be subject to a high degree of noise. For seven locations where this filtering process left very few OTUs (fewer than 900), we relaxed this criterion to use OTUs present in two or more plots (electronic supplementary material, table S1). Note that we did not use DESeq2 to test for statistical significance (as the program is often used) but exported the normalized log_2_-fold ratios for further analyses.

### (e) Phylogenetic conservation of responses at each location

To assess whether the response to each perturbation was phylogenetically conserved at an individual location, representative sequences of each OTU (the most abundant sequence within each OTU from the experiments) were aligned using the DECIPHER package [52]. A neighbour-joining (NJ) phylogenetic tree was inferred with bootstrap analysis (100 replicates) using the phangorn package [53]. We then applied a consenTRAIT analysis [4] (using the castor package [54]) to test whether an OTU's response to perturbation was related to the bacterial phylogeny.

The consenTRAIT algorithm identifies phylogenetic clades in which the direction of the response is conserved (consensus clades) and calculates the average depth of those clades from a phylogenetic tree. If the response is significantly conserved, then the average phylogenetic depth of those clades is greater than a distribution of randomly distributed responses. The consenTRAIT approach only considers binary traits (here, whether the response is positive or negative). A positive or negative response was assigned for each OTU on the NJ phylogenetic tree based on the log_2_-fold ratio exported from DESeq2. The tree was traversed from the root to the tips, recording the deepest nodes where more than 90% of the descending tips (OTUs) shared the same directional response (a ‘consensus’ clade). The genetic depth (the average distance of the node to its descending tips) and size (total number of the descending tips) of each consensus clade was calculated. The genetic depth of clades with a single descending tip (OTU) was calculated as half the branch length to the nearest neighbour as previously recommended [4]. Finally, the mean genetic depth, *τ*_D_, of the consensus clades sharing either positive or negative responses was calculated (electronic supplementary material, figure S2). To assess the statistical significance of phylogenetic conservation of responses, simulated *τ*_D_ values were calculated by randomizing the responses among the tips 1000 times. The probability of phylogenetic conservation (non-randomness) of the distribution of positive and negative responses was calculated as the fraction of simulated *τ*_D_ values that were greater than or equal to the observed *τ*_D_.

We used NJ trees for the consenTRAIT analysis, because the genetic scale of these trees roughly represents sequence dissimilarity. However, to consider whether our results were robust to the phylogenetic reconstruction method, we tested for a correlation between NJ and maximum-likelihood (ML) trees for each perturbation. A ML tree with 100 bootstrap replications was constructed with RAxML v8.0, using the GTR + Gamma distribution model [55] at the CIPRES science gateway (v. 3.3, http://www.phylo.org/index.php/).

### (f) Phylogenetic conservation of responses across locations

To assess whether the responses were context dependent, we identified 1364 (warming), 1557 (drought), 1284 (elevated CO_2_) and 1079 (P addition) OTUs that were present in three or more experimental locations. (Note these OTUs were from the pool of non-rare OTUs in each location; electronic supplementary material, table S1.) For each of these widespread OTUs, we averaged the response values across locations. This procedure treats the results of each location equally, regardless of differences in methods and sequencing effort. Unlike a typical diversity metric (e.g. a metric of richness or phylogenetic diversity), the response ratio parameter that we estimate for each taxon in a location should not be biased by sequencing effort, although it will presumably get more accurate with more sequencing. For each perturbation experiment, we then created a NJ tree of these widespread OTUs and performed the consenTRAIT analysis as above with this merged dataset.

Because the responses of widespread OTUs were significantly phylogenetically conserved, we next identified the taxonomy of clades whose response to perturbation was significantly more positive or negative than expected by chance using the RDP classifications with the SILVA database (version 132). Notably, this version of SILVA uses the Genome Taxonomy Database (GTDB), which classifies taxonomy based on monophyletic lineages and normalizes taxonomic ranks based on phylogenetic depth [56]. We then calculated the number of OTUs that had a positive or negative response at each phylum, class, order, family or genus level. We performed a two-tailed exact test [57] against the equal distribution of positive and negative responses within each taxonomic group. To compare these results with our previous study about bacterial responses to N addition where the older SILVA database (v. 128) was used [14], we re-identified the taxonomy of those clades with the new SILVA version.

## 3. Results

### (a) Overall bacterial community composition

After reanalysing the sequence data in a consistent way across all datasets, we first tested whether the perturbations altered overall community composition. Bacterial community composition had a mixed response to the perturbation types. The perturbations significantly altered bacterial composition (PERMANOVA; *p* < 0.05) in fewer than 50% of the locations (11 of 26 locations), although if locations of marginal significance are included (*p* < 0.1), this number increases to 60% (16 of 26 locations, table 2). Where composition significantly shifted, the perturbation explained between 5.8 and 28.6% of compositional variation.

### (b) Phylogenetic conservation of bacterial responses at individual locations

In contrast with overall composition, bacterial responses were significantly conserved within the majority of locations across all perturbation types. For instance, the responses to soil warming were conserved at all six locations; the mean genetic depth (*τ*_D_) of the consensus clades (the clades in which more than 90% of the descendant OTUs show the same response direction) for both or either of the responses (positive and negative) was greater than expected given a randomized distribution of responses (permutation test; *p* < 0.05, table 2). The responses to the other perturbations were phylogenetically patterned in 6 of 7, 5 of 6 and 3 of 6 locations, for drought, elevated CO_2_ and P addition, respectively. The response to the single soil liming experiment was also phylogenetically patterned (table 2). As we found previously [14], the consenTRAIT results were robust to the phylogenetic reconstruction method (neighbour-joining or maximum-likelihood), as expected from the high correlation between trees for the different locations and perturbation types (electronic supplementary material, table S2). Notably, the phylogenetic analysis was more sensitive than the community analysis; the locations that displayed overall shifts in bacterial composition were a subset of those that showed significant phylogenetic conservation. This discrepancy likely arises because the phylogenetic analysis gives equal weight to all taxa, whereas the community analysis primarily considers the most abundant taxa.

**Figure 2.**
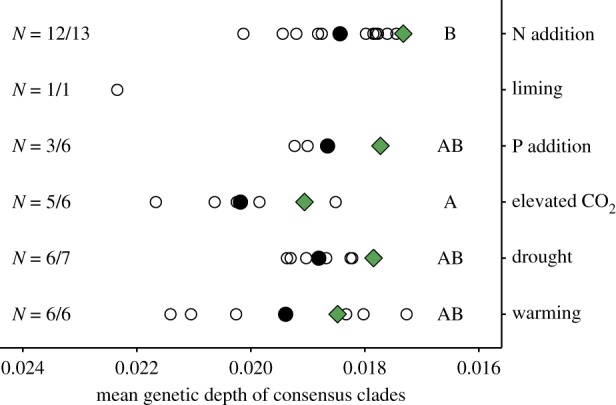
Mean genetic depth (*τ*_D_) of consensus clades as calculated with the consenTRAIT algorithm. Open circles indicate *τ*_D_ at each location. Filled circles indicate the average *τ*_D_ at individual locations. Filled (green) diamonds indicate *τ*_D_ of widespread OTUs present in at least three locations. For comparison, the *τ*_D_ values from N addition experiments from [14] are also plotted. The number of locations where the responses were significantly phylogenetically patterned (positive and/or negative) out of the total locations is shown on the left margin (table 2). Different letters on the right margin indicate significant differences (*p* < 0.05) in the mean *τ*_D_ between perturbations based on one-way ANOVA with Tukey's honestly significant difference. (Online version in colour.)

While the responses to the perturbations were phylogenetically conserved, there was less evidence that the degree of conservation varied by perturbation type, in contrast with our first hypothesis (figure 1*b*). The mean depth of the consensus clades responding positively or negatively (*τ*_D_) ranged from 0.017 to 0.024 across all perturbations and all locations (table 2), equivalent to an average sequence dissimilarity in the 16S rRNA gene amplicon of 3.4–4.8% among OTUs. These values differed among the perturbation types (one-way ANOVA; *p* = 0.05, figure 2). This difference was driven by the deeper level of conservation of the elevated CO_2_ response than of the N addition response (*post hoc* Tukey test: *p* = 0.03), whereas the depth of conservation of responses to N addition, P addition, drought and warming experiments substantially overlapped. The soil liming experiment showed the deepest response of any study location, but as the only experiment of its type, we excluded it from the statistical test.

### (c) Context dependence of the bacterial responses

**Figure 3.**
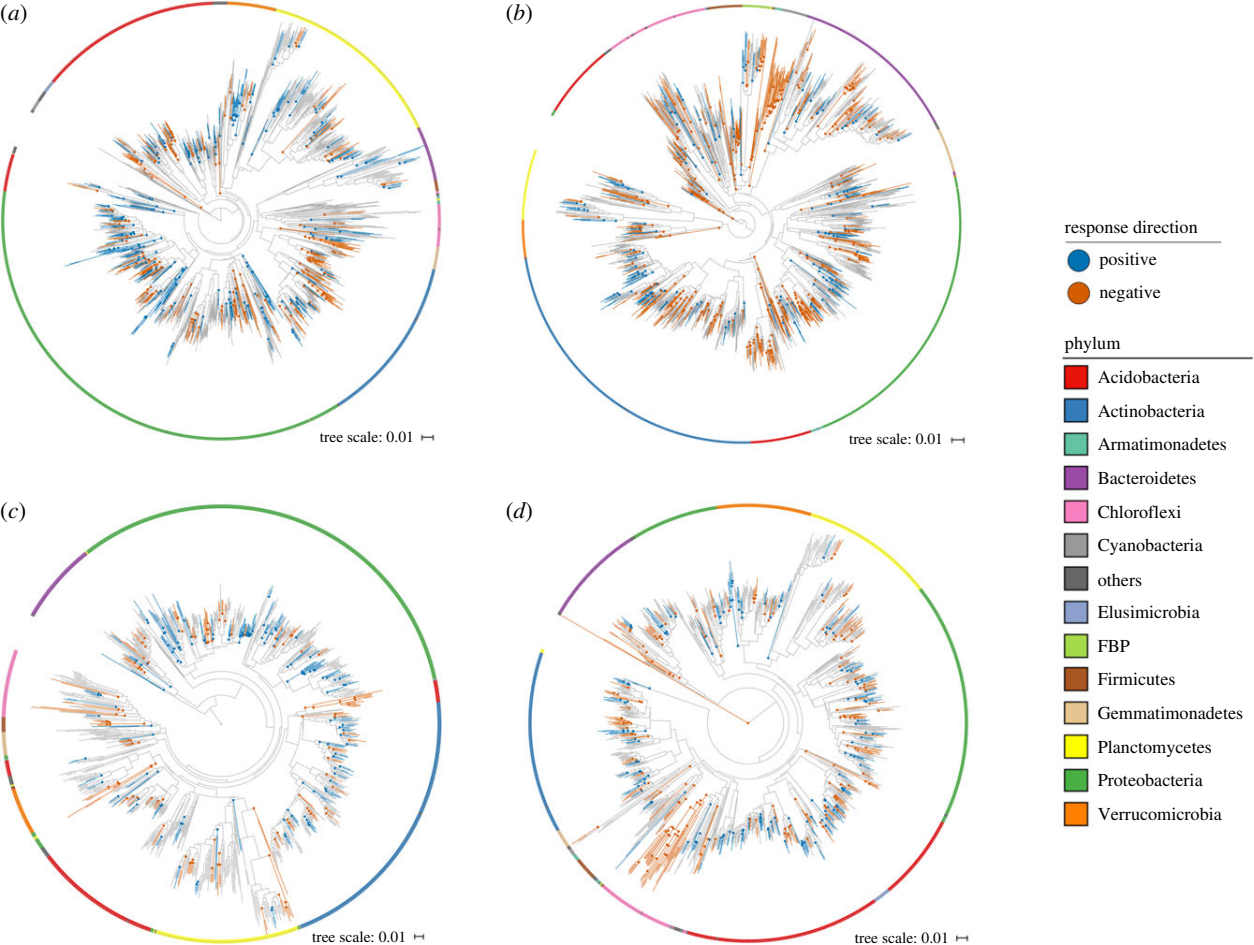
Phylogenetic distribution of the averaged responses to (*a*) soil warming, (*b*) drought, (*c*) CO_2_ elevation and (*d*) P addition of widespread OTUs (i.e. present at three locations or more). Coloured nodes and lineages show the consensus clades in which more than 90% of the descendant OTUs show the same response direction (blue, positive response; red, negative response). Note that all OTUs in the tree respond, but only consensus clades are coloured for clarity. The outer ring shows the phylum-level taxonomy of OTUs determined using the RDP classifier trained on the SILVA database.

To investigate the context dependence of the responses, we next quantified the average responses of widespread OTUs across locations. Widespread OTUs (present in at least three locations) accounted for more than 50% of the sequences at all locations except one (Bouskill_2) (electronic supplementary material, figure S3). The average responses of these widespread OTUs were also phylogenetically conserved for all perturbations (excluding liming; permutation test; *p* < 0.05, table 2 and figure 3), indicating that the context of the particular location and experiment did not overshadow the phylogenetic signal of the responses observed at individual locations. However, contrary to our second hypothesis (figure 1*c*), the degree of context dependence did not vary by perturbation. For all perturbations, the depth (average *τ*_D_ of positive and negative responses) of the clades conserved across locations was exactly 0.001 smaller (only 0.2% difference in 16S rDNA sequence) than the mean of the individual locations (diamond symbols in figure 2).

### (d) Correlations of responses among perturbation types

**Figure 4.**
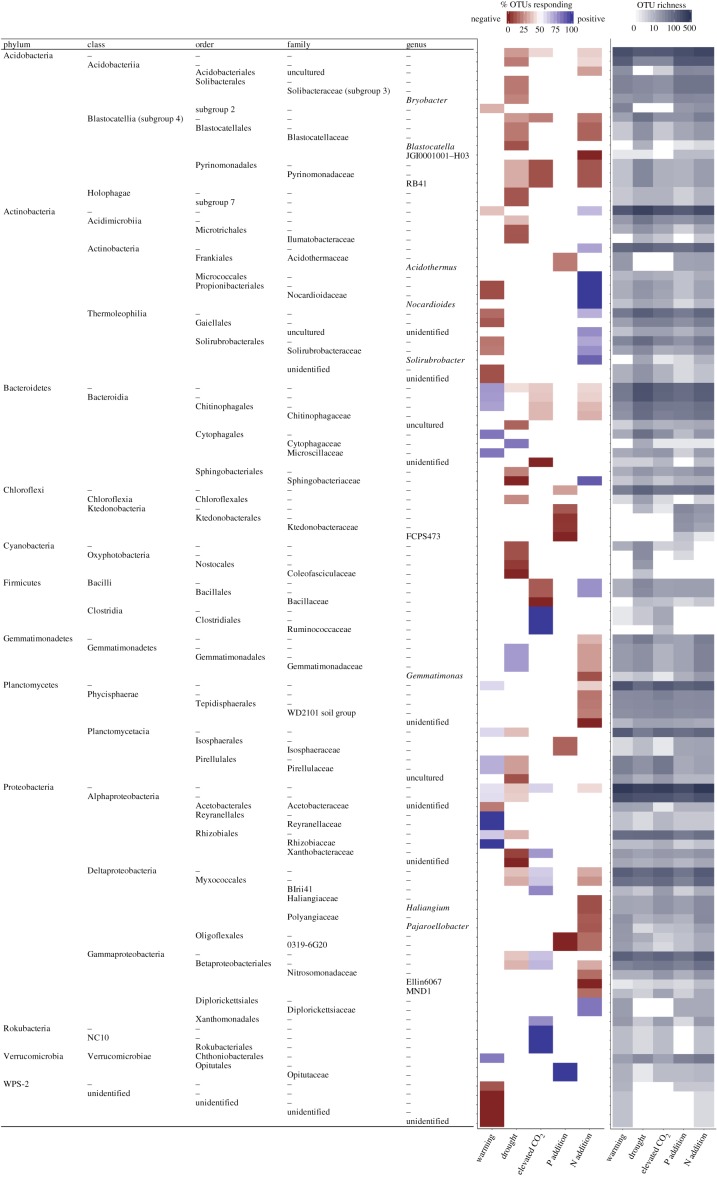
Taxonomic groupings whose response was positive or negative. Responses are coloured when they display significantly more positive or negative responses than expected by chance (two-tailed exact test; *p* < 0.05). The per cent of OTUs responding positively (blue) or negatively (red) is plotted in the heatmap to the left. The number of OTUs are plotted in the heatmap to the right.

Finally, while the average genetic depth of consensus clades (*τ*_D_) was relatively shallow for all perturbations, some deep clades (defined by the GTDB taxonomy) responded consistently to some perturbations. For instance, all major phyla present responded consistently to at least one type of perturbation; the direction of the OTU responses within the phylum was significantly more negative or positive rather than random (figure 4; electronic supplementary material, table S3). In particular, more than 80% of OTUs within the phyla Cyanobacteria, Rokubacteria and WPS-2 responded consistently to a perturbation (figure 4; electronic supplementary material, table S3). As one would expect, these patterns were generally even stronger at the class and order levels; for example, the classes Acidobacteriia, Blastocatellia and Holophagae appear to be driving the overall negative response to drought within the phylum Acidobacteria.

Contrary to our third hypothesis (figure 1*d*), however, there was little evidence that a clade that responds positively or negatively to one type of perturbation will also respond to other perturbations. In fact, there were relatively few instances where the same taxonomic group responded consistently to more than one perturbation type (figure 4). This result did not seem to be driven by differences in the taxonomic diversity present among the experiments; OTU richness within the taxonomic groups was largely similar across perturbation types (figure 4). For example, many OTUs in the Planctomycetes and Proteobacteria were present in more than one perturbation, but most of the taxonomic groups within these phyla showed consistent responses to only one type of perturbation.

Among the taxonomic groups that responded significantly to two or more perturbations, the responses were often in both directions, showing a positive response to some perturbations and a negative response to others. For instance, the orders Propionibacteriales and Solirubrobacterales within the Actinobacteria responded negatively to warming and positively to N addition. Similarly, the family Sphingobacteriaceae (phylum Bacteroidetes) responded in opposing directions to drought (negative) and N addition (positive). The only group that seemed to defy this trend was the phylum Acidobacteria. As a whole, the phylum responded negatively to drought, elevated CO_2_ and N addition.

## 4. Discussion

Our re-analysis of field experiment data provides several indications that phylogenetic information can be used to predict the response of soil bacteria to global changes. Overall, bacterial responses were phylogenetically conserved within the majority of locations across all perturbation types. More specifically, the depth of conservation of these responses did not vary much by perturbation type, inconsistent with our first hypothesis. Further, the degree of context dependence did not depend on the type of perturbation, countering our second hypothesis, but indicating that the results from a variety of perturbation experiments in one location can help predict bacterial community responses in other locations.

Contrary to our third hypothesis, however, there was little evidence of clades that were generally sensitive to perturbations. Indeed, at a broad taxonomic level, most groups responded consistently to only one or two types of perturbations, suggesting that responses to different perturbations are generally due to different traits rather than common traits that broadly increase or decrease a clade's sensitivity to environmental change. A notable example is the response to N and P addition. Although one might hypothesize that some shared traits might explain the response to nutrient addition generally, only one family within the order Oligoflexales responded consistently (negatively) to both treatments. This result is consistent with previous studies that found that different taxa (OTUs) from the same community responded to different perturbations (e.g. heat shock versus cold shock [28] and warming versus drought [29]). Our study suggests that this trend might hold true for broader taxonomic groups across a range of locations and perturbations.

However, the lack of a correlation between a taxonomic group's sensitivity to one perturbation versus its response to others might ultimately make predictions easier. Further, the result that few taxonomic groups showed consistent responses to more than one perturbation might mean that the interactive effects of multiple global changes on microbial composition might be minimal. On the other hand, the evidence of legacy effects on microbial composition, where the community response to one perturbation influences the response to future perturbations [11,28,35,58], perhaps suggests the opposite. Additional research on the phylogenetic patterns of responses to co-occurring perturbations is therefore needed, as future environmental changes will not occur in isolation [59].

The phylogenetic patterns described above provide several insights into the traits underlying the responses of soil bacteria in the face of a perturbation [60–62]). First, the depth of phylogenetic conservation of all responses fell within a narrow range. The average depth across perturbations (*τ*_D_ = 0.018–0.020) corresponds to a 3.6–4.0% divergence in the 16S rRNA gene amplicon, or approximately the level of a bacterial genus [63]. The one exception was a study that manipulated pH by liming (*τ*_D_ = 0.022), and pH preference appears to be quite deeply conserved relative to other traits [3]. All of these values are relatively shallow compared with traits such as methanogenesis (*τ*_D_ = 0.071), but much more conserved than organic phosphorus acquisition or carbon substrate utilization, for example. We expect that the response to a perturbation would be governed by a suite of traits that vary in conservation depth. For instance, the response to drought might involve a deeply conserved trait such as spore formation and cell wall type [18] as well as shallower traits such as salt tolerance or biosynthesis of organic osmotic solutes [64].

The patterns in phylogenetic responses also suggest that there is not a common suite of traits involved in responding to most perturbations, as indicated by the lack of correlation in responses among the types of experiments. Instead, the underlying traits responsible for a conserved response seem to be specific to each perturbation type, even though the degree of conservation among the bacterial responses was similar. It is also important to note that the responses to some perturbations might not be due to direct effects of changes in abiotic conditions, but to indirect effects of changes in plant communities and soil and litter chemistry. For instance, warming might increase nutrient availability through the enhanced supply of plant root exudates or decrease nutrient availability through the drying of soil and suppression of litter decomposition [65,66]. Such indirect effects might also explain why it might be easier to observe phylogenetic patterns of bacterial responses in these experimental studies versus along biogeographic gradients [67].

Finally, the phylogenetic responses provide clues about the traits of rare and understudied taxa. For instance, the uncultivated candidate phylum WPS-2, first observed in polluted soil via clone library analysis [68], responded negatively to warming. WPS-2 has a global distribution but most samples containing WPS-2 with high abundance were collected typically from cold environments [69], such as Antarctica soil [70] and Greenland ice [71]. The uncultivated candidate phylum Rokubacteria, first observed in an alpine meadow via clone library analysis [72], responded positively to elevated CO_2_. Metagenomic assemblies suggest that the order Rokubacteriales has the potential for a versatile, mixotrophic metabolism [73]. Elevated CO_2_ might increase the release of root exudates to soil, enhance nutrient availability and shift the ecological strategy of the soil bacterial community to a higher contribution of fast-growing r-selected taxa [27].

Of course, there are notable limitations to these results, and these caveats point to future areas of research. Perhaps the most importantly, we could only obtain sequence data from a small number of experiments for each type of perturbation. These experimental locations represent a limited number of ecosystems and geographic regions. Indeed, some experiments were located quite close to one another, and all of the elevated CO_2_ and P addition experiments were conducted in grasslands. Thus, the degree to which the bacterial responses are context dependent might increase with the inclusion of additional experiments from a broader range of soil biomes. By contrast, a wider representation of soil communities would increase the degree of phylogenetic representation across bacterial phylogeny and improve our ability to predict the responses of particular taxa or clades to particular perturbations.

## 5. Conclusion

The enormous diversity of soil bacteria would seem to overwhelm attempts to make detailed predictions about their responses to global change. Nevertheless, this study provides additional evidence that efforts to compile databases of microbial traits [74], classify responses to within experiments [2] and develop new statistical methods [75] make this a tractable goal. A critical next step, however, is to connect shifts in community composition to the functional processes soil bacteria carry out. Many potential functions (as predicted through genomic traits) are phylogenetically conserved [4,74], and new techniques estimating taxon-specific process rates find that these measures are also phylogenetically patterned [10,19]. Finally, recent evidence suggests that phylogeny has a stronger effect than the environment on bacterial processes such as growth rate and carbon assimilation [76]. This result suggests that, like their responses in abundance, bacterial process rates might not be overly context dependent. In this case, phylogenetic patterns of bacterial responses and their functional traits could be combined to predict how global changes will alter ecosystem functioning, as has been proposed for both microbial and plant communities [62,77].

## Supplementary Material

Supplementary File 1

## Supplementary Material

Supplementary File 2

## Data accessibility

Information about sequence data accessibility is provided in electronic supplementary material, table S1. Our reanalysis of the datasets is available at https://github.com/kazuo-isobe/phylogenetic-conservation-global-change and from the authors upon request.

## References

[1] Shade A et al*.* 2012 Fundamentals of microbial community resistance and resilience. Front. Microbiol. 3, 417 (10.3389/fmicb.2012.00417) 23267351 PMC3525951

[2] Rocca JD, Simonin M, Blaszczak JR, Ernakovich JG, Gibbons SM, Midani FS, Washburne AD 2019 The Microbiome Stress Project: toward a global meta-analysis of environmental stressors and their effects on microbial communities. Front. Microbiol. 9, 3272 (10.3389/fmicb.2018.03272) 30687263 PMC6335337

[3] Martiny JBH, Jones SE, Lennon JT, Martiny AC 2015 Microbiomes in light of traits: a phylogenetic perspective. Science 350, aac9323 (10.1126/science.aac9323) 26542581

[4] Martiny AC, Treseder K, Pusch G 2013 Phylogenetic conservatism of functional traits in microorganisms. ISME J. 7, 830–838. (10.1038/ismej.2012.160) 23235290 PMC3603392

[5] Mouquet N et al*.* 2012 Ecophylogenetics: advances and perspectives. Biol. Rev. 87, 769–785. (10.1111/j.1469-185X.2012.00224.x) 22432924

[6] Philippot L, Andersson SGE, Battin TJ, Prosser JI, Schimel JP, Whitman WB, Hallin S 2010 The ecological coherence of high bacterial taxonomic ranks. Nat. Rev. Microbiol. 8, 523–529. (10.1038/nrmicro2367) 20531276

[7] Goberna M, Verdú M 2018 Phylogenetic-scale disparities in the soil microbial diversity–ecosystem functioning relationship. ISME J. 12, 2152–2162. (10.1038/s41396-018-0162-5) 29880911 PMC6092336

[8] Doolittle WF 1999 Lateral genomics. Trends Biochem. Sci. 24, M5–M8. (10.1016/S0968-0004(99)01471-1) 10611671

[9] Doolittle WF 1999 Phylogenetic classification and the universal tree. Science 284, 2124–2128. (10.1126/science.284.5423.2124) 10381871

[10] Placella SA, Brodie EL, Firestone MK 2012 Rainfall-induced carbon dioxide pulses result from sequential resuscitation of phylogenetically clustered microbial groups. Proc. Natl Acad. Sci. USA 109, 10 931–10 936. (10.1073/pnas.1204306109) PMC3390866 22715291

[11] Evans SE, Wallenstein MD 2014 Climate change alters ecological strategies of soil bacteria. Ecol. Lett. 17, 155–164. (10.1111/ele.12206) 24261594

[12] Morrissey EM et al*.* 2016 Phylogenetic organization of bacterial activity. ISME J. 10, 2336–2340. (10.1038/ismej.2016.28) 26943624 PMC4989319

[13] Amend AS, Martiny AC, Allison SD, Berlemont R, Goulden ML, Lu Y, Treseder KK, Weihe C, Martiny JBH 2016 Microbial response to simulated global change is phylogenetically conserved and linked with functional potential. ISME J. 10, 109–118. (10.1038/ismej.2015.96) 26046258 PMC4681869

[14] Isobe K, Allison SD, Khalili B, Martiny AC, Martiny JBH 2019 Phylogenetic conservation of bacterial responses to soil nitrogen addition across continents. Nat. Commun. 10, 2499 (10.1038/s41467-019-10390-y) 31175309 PMC6555827

[15] Tilman D et al*.* 2001 Forecasting agriculturally driven global environmental change. Science 292, 281–284. (10.1126/science.1057544) 11303102

[16] Cavicchioli R et al*.* 2019 Scientists' warning to humanity: microorganisms and climate change. Nat. Rev. Microbiol. 7, 451–459. (10.1038/s41579-019-0222-5) PMC7136171 31213707

[17] Song J et al*.* 2019 A meta-analysis of 1,119 manipulative experiments on terrestrial carbon-cycling responses to global change. Nat. Ecol. Evol. 3, 1309–1320. (10.1038/s41559-019-0958-3) 31427733

[18] Barberán A, Caceres Velazquez H, Jones S, Fierer N 2017 Hiding in plain sight: mining bacterial species records for phenotypic trait information. mSphere 2, e00237-17 (10.1128/mSphere.00237-17) 28776041 PMC5541158

[19] Morrissey EM, Mau RL, Schwartz E, Koch BJ, Hayer M, Hungate BA 2018 Taxonomic patterns in the nitrogen assimilation of soil prokaryotes. Environ. Microbiol. 20, 1112–1119. (10.1111/1462-2920.14051) 29411496

[20] Schimel J, Balser TC, Wallenstein M 2007 Microbial stress-response physiology and its implications for ecosystem function. Ecology 88, 1386–1394. (10.1890/06-0219) 17601131

[21] Bouskill NJ et al*.* 2016 Belowground response to drought in a tropical forest soil. I. Changes in microbial functional potential and metabolism. Front. Microbiol. 7, 525 (10.3389/fmicb.2016.00525) 27148214 PMC4837414

[22] Wood JM 2015 Bacterial responses to osmotic challenges. J. Gen. Physiol. 145, 381–388. (10.1085/jgp.201411296) 25870209 PMC4411257

[23] Allison SD, Goulden ML 2017 Consequences of drought tolerance traits for microbial decomposition in the DEMENT model. Soil Biol. Biochem. 107, 104–113. (10.1016/j.soilbio.2017.01.001)

[24] Leff JW et al*.* 2015 Consistent responses of soil microbial communities to elevated nutrient inputs in grasslands across the globe. Proc. Natl Acad. Sci. 112, 10 967–10 972. (10.1073/pnas.1508382112) 26283343 PMC4568213

[25] Yao Q et al*.* 2018 Community proteogenomics reveals the systemic impact of phosphorus availability on microbial functions in tropical soil. Nat. Ecol. Evol. 2, 499–509. (10.1038/s41559-017-0463-5) 29358607

[26] Classen AT, Sundqvist MK, Henning JA, Newman GS, Moore JAM, Cregger MA, Moorhead LC, Patterson CM 2015 Direct and indirect effects of climate change on soil microbial and soil microbial-plant interactions: what lies ahead? Ecosphere 6, art130 (10.1890/ES15-00217.1)

[27] Blagodatskaya E, Blagodatsky S, Dorodnikov M, Kuzyakov Y 2010 Elevated atmospheric CO_2_ increases microbial growth rates in soil: results of three CO_2_ enrichment experiments. Glob. Chang. Biol. 16, 836–848. (10.1111/j.1365-2486.2009.02006.x)

[28] Jurburg SD, Nunes I, Brejnrod A, Jacquiod S, Priemé A, Sørensen SJ, Van Elsas JD, Salles JF 2017 Legacy effects on the recovery of soil bacterial communities from extreme temperature perturbation. Front. Microbiol. 8, 1832 (10.3389/fmicb.2017.01832) 28993764 PMC5622210

[29] Zhang K, Shi Y, Jing X, He J-S, Sun R, Yang Y, Shade A, Chu H 2016 Effects of short-term warming and altered precipitation on soil microbial communities in alpine grassland of the Tibetan plateau. Front. Microbiol. 7, 1032 (10.3389/fmicb.2016.01032) 27446064 PMC4927576

[30] Goberna M, Navarro-Cano JA, Valiente-Banuet A, García C, Verdú M 2014 Abiotic stress tolerance and competition-related traits underlie phylogenetic clustering in soil bacterial communities. Ecol. Lett. 17, 1191–1201. (10.1111/ele.12341) 25130277

[31] Che R et al*.* 2018 Long-term warming rather than grazing significantly changed total and active soil procaryotic community structures. Geoderma 316, 1–10. (10.1016/j.geoderma.2017.12.005)

[32] DeAngelis KM, Pold G, Topçuoglu BD, van Diepen LTA, Varney RM, Blanchard JL, Melillo J, Frey SD. 2015 Long-term forest soil warming alters microbial communities in temperate forest soils. Front. Microbiol. 6, 104 (10.3389/fmicb.2015.00104) 25762989 PMC4327730

[33] Waghmode TR, Chen S, Li J, Sun R, Liu B, Hu C 2018 Response of nitrifier and denitrifier abundance and microbial community structure to experimental warming in an agricultural ecosystem. Front. Microbiol. 9, 474 (10.3389/fmicb.2018.00474) 29593703 PMC5861319

[34] Bastida F et al*.* 2017 Differential sensitivity of total and active soil microbial communities to drought and forest management. Glob. Chang. Biol. 23, 4185–4203. (10.1111/gcb.13790) 28614633

[35] Bouskill NJ, Lim HC, Borglin S, Salve R, Wood TE, Silver WL, Brodie EL 2013 Pre-exposure to drought increases the resistance of tropical forest soil bacterial communities to extended drought. ISME J. 7, 384–394. (10.1038/ismej.2012.113) 23151641 PMC3554394

[36] Fernandes VMC, Machado de Lima NM, Roush D, Rudgers J, Collins SL, Garcia-Pichel F 2018 Exposure to predicted precipitation patterns decreases population size and alters community structure of cyanobacteria in biological soil crusts from the Chihuahuan Desert. Environ. Microbiol. 20, 259–269. (10.1111/1462-2920.13983) 29124873

[37] Deng Y et al*.* 2012 Elevated carbon dioxide alters the structure of soil microbial communities. Appl. Environ. Microbiol. 78, 2991–2995. (10.1128/AEM.06924-11) 22307288 PMC3318805

[38] Raut S, Polley HW, Fay PA, Kang S 2018 Bacterial community response to a preindustrial-to-future CO_2_ gradient is limited and soil specific in Texas prairie grassland. Glob. Chang. Biol. 24, 5815–5827. (10.1111/gcb.14453) 30230661

[39] Xia W, Jia Z, Bowatte S, Newton PCD 2017 Impact of elevated atmospheric CO_2_ on soil bacteria community in a grazed pasture after 12-year enrichment. Geoderma 285, 19–26. (10.1016/j.geoderma.2016.09.015)

[40] Yang S et al*.* 2019 Long-term elevated CO_2_ shifts composition of soil microbial communities in a Californian annual grassland, reducing growth and N utilization potentials. Sci. Total Environ. 652, 1474–1481. (10.1016/j.scitotenv.2018.10.353) 30586832

[41] Guo A, Ding L, Tang Z, Zhao Z, Duan G 2019 Microbial response to CaCO_3_ application in an acid soil in southern China. J. Environ. Sci. 79, 321–329. (10.1016/j.jes.2018.12.007) 30784455

[42] Goulding KWT 2016 Soil acidification and the importance of liming agricultural soils with particular reference to the United Kingdom. Soil Use Manag. 32, 390–399. (10.1111/sum.12270) 27708478 PMC5032897

[43] Loman NJ, Misra RV, Dallman TJ, Constantinidou C, Gharbia SE, Wain J, Pallen MJ 2012 Performance comparison of benchtop high-throughput sequencing platforms. Nat. Biotechnol. 30, 434–439. (10.1038/nbt.2198) 22522955

[44] Martin M 2011 Cutadapt removes adapter sequences from high-throughput sequencing reads. EMBnet.journal 17, 10 (10.14806/ej.17.1.200)

[45] Edgar RC 2013 UPARSE: highly accurate OTU sequences from microbial amplicon reads. Nat. Methods 10, 996–998. (10.1038/nmeth.2604) 23955772

[46] Wang Q, Garrity GM, Tiedje JM, Cole JR 2007 Naive Bayesian classifier for rapid assignment of rRNA sequences into the new bacterial taxonomy. Appl. Environ. Microbiol. 73, 5261–5267. (10.1128/AEM.00062-07) 17586664 PMC1950982

[47] Walters W, Hyde ER, Berg-Lyons D, Ackermann G, Humphrey G, Parada A, Gilbert JA, Jansson JK 2015 Transcribed spacer marker gene primers for microbial community surveys. mSystems 1, e0009-15 (10.1128/mSystems.00009-15) PMC5069754 27822518

[48] Callahan BJ, McMurdie PJ, Holmes SP 2017 Exact sequence variants should replace operational taxonomic units in marker-gene data analysis. ISME J. 11, 2639–2643. (10.1038/ismej.2017.119) 28731476 PMC5702726

[49] Oksanen J, Blanchet FG, Kindt R, Legendre P, Minchin PR, Hara RBO, Simpson GL, Solymos P, Stevens MHH 2017 *vegan: Community Ecology Package* See http://CRAN.R-project.org/package=vegan (accessed 1 June 2019).

[50] Love MI, Huber W, Anders S 2014 Moderated estimation of fold change and dispersion for RNA-seq data with DESeq2. Genome Biol. 15, 550 (10.1186/s13059-014-0550-8) 25516281 PMC4302049

[51] McMurdie PJ, Holmes S 2013 phyloseq: an R package for reproducible interactive analysis and graphics of microbiome census data. PLoS ONE 8, e61217 (10.1371/journal.pone.0061217) 23630581 PMC3632530

[52] Wright ES 2016 Using DECIPHER v2.0 to analyze big biological sequence data in R. R J. 8, 352–359. (10.32614/RJ-2016-025)

[53] Schliep KP 2011 phangorn: phylogenetic analysis in R. Bioinformatics 27, 592–593. (10.1093/bioinformatics/btq706) 21169378 PMC3035803

[54] Louca S, Doebeli M 2018 Efficient comparative phylogenetics on large trees. Bioinformatics 34, 1053–1055. (10.1093/bioinformatics/btx701) 29091997

[55] Stamatakis A 2014 RAxML version 8: a tool for phylogenetic analysis and post-analysis of large phylogenies. Bioinformatics 30, 1312–1313. (10.1093/bioinformatics/btu033) 24451623 PMC3998144

[56] Parks DH, Chuvochina M, Waite DW, Rinke C, Skarshewski A, Chaumeil P-A, Hugenholtz P 2018 A standardized bacterial taxonomy based on genome phylogeny substantially revises the tree of life. Nat. Biotechnol. 36, 996–1004. (10.1038/nbt.4229) 30148503

[57] McDonald JH 2015 Handbook of biological statistics, 3rd edn Baltimore, MD: Sparky House Publishing.

[58] Hawkes CV, Keitt TH 2015 Resilience vs. historical contingency in microbial responses to environmental change. Ecol. Lett. 18, 612–625. (10.1111/ele.12451) 25950733

[59] Garcia RA, Cabeza M, Rahbek C, Araújo MB 2014 Multiple dimensions of climate change and their implications for biodiversity. Science 344, 12475979 (10.1126/science.1247579) 24786084

[60] Lavorel S, Garnier E 2002 Predicting changes in community composition and ecosystem functioning from plant traits: revisiting the Holy Grail. Funct. Ecol. 16, 545–556. (10.1046/j.1365-2435.2002.00664.x)

[61] Suding KN et al*.* 2008 Scaling environmental change through the community-level: a trait-based response-and-effect framework for plants. Glob. Chang. Biol. 14, 1125–1140. (10.1111/j.1365-2486.2008.01557.x)

[62] Allison SD, Martiny JBH 2008 Resistance, resilience, and redundancy in microbial communities. Proc. Natl Acad. Sci. 105, 11 512–11 519. (10.1073/pnas.0801925105) 18695234 PMC2556421

[63] Konstantinidis KT, Tiedje JM 2005 Towards a genome-based taxonomy for prokaryotes. J. Bacteriol. 187, 6258–6264. (10.1128/JB.187.18.6258-6264.2005) 16159757 PMC1236649

[64] Oren A 2008 Microbial life at high salt concentrations: phylogenetic and metabolic diversity. Saline Systems 4, 2 (10.1186/1746-1448-4-2) 18412960 PMC2329653

[65] Walker TWN, Kaiser C, Strasser F, Herbold CW, Leblans NIW, Woebken D, Janssens IA, Sigurdsson BD, Richter A 2018 Microbial temperature sensitivity and biomass change explain soil carbon loss with warming. Nat. Clim. Chang. 8, 885–889. (10.1038/s41558-018-0259-x) 30288176 PMC6166784

[66] Alatalo JM, Jägerbrand AK, Juhanson J, Michelsen A, L'Uptáčik P 2017 Impacts of twenty years of experimental warming on soil carbon, nitrogen, moisture and soil mites across alpine/subarctic tundra communities. Scient. Rep. 7, 44489 (10.1038/srep44489) PMC5353735 28295022

[67] Oliverio AM, Bradford MA, Fierer N 2017 Identifying the microbial taxa that consistently respond to soil warming across time and space. Glob. Chang. Biol. 23, 2117–2129. (10.1111/gcb.13557) 27891711

[68] Nogales B, Moore ERB, Llobet-Brossa E, Rossello-Mora R, Amann R, Timmis KN 2001 Combined use of 16S ribosomal DNA and 16S rRNA to study the bacterial community of polychlorinated biphenyl-polluted soil. Appl. Environ. Microbiol. 67, 1874–1884. (10.1128/AEM.67.4.1874-1884.2001) 11282645 PMC92809

[69] Ward LM, Cardona T, Holland-Moritz H 2019 Evolutionary implications of anoxygenic phototrophy in the bacterial phylum *Candidatus* Eremiobacterota (WPS-2). Front. Microbiol. 10, 1658 (10.3389/fmicb.2019.01658) 31396180 PMC6664022

[70] Ji M et al*.* 2017 Atmospheric trace gases support primary production in Antarctic desert surface soil. Nature 552, 400–403. (10.1038/nature25014) 29211716

[71] Stibal M, Schostag M, Cameron KA, Hansen LH, Chandler DM, Wadham JL, Jacobsen CS 2015 Different bulk and active bacterial communities in cryoconite from the margin and interior of the Greenland ice sheet. Environ. Microbiol. Rep. 7, 293–300. (10.1111/1758-2229.12246) 25405749

[72] Lipson DA, Schmidt SK 2004 Seasonal changes in an alpine soil bacterial community in the Colorado Rocky Mountains. Appl. Environ. Microbiol. 70, 2867–2879. (10.1128/AEM.70.5.2867) 15128545 PMC404381

[73] Becraft ED et al*.* 2017 Rokubacteria: genomic giants among the uncultured bacterial phyla. Front. Microbiol. 8, 2264 (10.3389/fmicb.2017.02264) 29234309 PMC5712423

[74] Mendler K, Chen H, Parks DH, Lobb B, Hug LA, Doxey AC 2019 AnnoTree: visualization and exploration of a functionally annotated microbial tree of life. Nucleic Acids Res. 47, 4442–4448. (10.1093/nar/gkz246) 31081040 PMC6511854

[75] Washburne AD, Silverman JD, Leff JW, Bennett DJ, Darcy JL, Mukherjee S, Fierer N, David LA 2017 Phylogenetic factorization of compositional data yields lineage-level associations in microbiome datasets. PeerJ 5, e2969 (10.7717/peerj.2969) 28289558 PMC5345826

[76] Morrissey EM et al*.* 2019 Evolutionary history constrains microbial traits across environmental variation. Nat. Ecol. Evol. 3, 1064–1069. (10.1038/s41559-019-0918-y) 31209289

[77] Díaz S, Purvis A, Cornelissen JHC, Mace GM, Donoghue MJ, Ewers RM, Jordano P, Pearse WD 2013 Functional traits, the phylogeny of function, and ecosystem service vulnerability. Ecol. Evol. 3, 2958–2975. (10.1002/ece3.601) 24101986 PMC3790543

