# Supplementary material for: Phylogenetic conservation of soil bacterial responses to simulated global changes
Source: Philos Trans R Soc Lond B Biol Sci. 2020 Mar 23;375(1798):20190242. doi: 10.1098/rstb.2019.0242 (PMC7133522; doi:10.1098/rstb.2019.0242)
Supplement: Supplementary File 1 [file rstb20190242supp1.pdf]

Table S1. Data availability, additional sample details of the study locations, and number of sequence counts and OTUs used in this study.

Perturbation	Locations	Sequence/metadata availability	Samples of original study	Sequence region of original study	Quality-filtered sequence count (average per plot)	No. OTUs before removing rare OTUs	No. OTUs after removing rare OTUs
Warming	Che [1]	NCBI under ID PRJNA417160	Grazing and non-grazing grasslands	V4-5	320,865 (20,054)	4,639	2,046*
	DeAngelis_1 [2]	NCBI under ID PRJNA242868	Swan Plots/ A horizon	V4	122,603 (15,325)	1,611	1,138**
	DeAngelis_2 [2]	NCBI under ID PRJNA242868	Barre Woods/ A horizon	V4	120,194 (15,024)	1,492	1,062**
	DeAngelis_3 [2]	NCBI under ID PRJNA242868	Prospect Hill/ A horizon	V4	116,007 (14,500)	1,403	1,046**
	Waghmode [3]	Provided by the author(s)	High and low irrigated croplands	V3–V4	208,046 (17,337)	4,280	2,298*
	Zhang [4]	Provided by the author(s)	Warming and control plots	V4-5	46,198 (3,849)	2,110	1,372*
Drought	Bastida_1 [5]	Provided by the author(s)	Un-thinned forest	V4	162,405 (13,533)	4,548	1,631*
	Bastida_2 [5]	Provided by the author(s)	Thinned forest	V4	158,512 (13,209)	4,721	1,698*
	Bouskill_1 [6]	Provided by the author(s)	All rainfall exclusion and control plots	V4-5	13,538 (2,256)	2,061	934**
	Bouskill_2	Provided by the author(s)	All rainfall exclusion and control plots	V4-5	298,200 (9,940)	2,032	1,730**
	Fernandes_1 [7]	Provided by the author(s)	site_Black	V4	1,044,733 (52,236)	6,787	1,903*
	Fernandes_2 [7]	Provided by the author(s)	site_Blue	V4	1,319,766 (65,988)	8,816	2,453*
	Zhang [4]	Provided by the author(s)	drought and control plots	V4-5	68,345 (5,695)	2,053	1,612
Elevated CO_2	Deng [8]	Provided by the author(s)	All elevated CO_2 and control plots	V4-5	16,207 (675)	1,896	1,077**
	Raut_1 [9]	NCBI under ID PRJNA416942	Clay soils/ 380-500 ppm (treatment), 250-380 ppm (control)	V4-5	466,527 (17,278)	7,446	1,771*
	Raut_2 [9]	NCBI under ID PRJNA416942	Silty loam soils/ 380-500 ppm (treatment), 250-380 ppm (control)	V4-5	687,559 (21,486)	8,118	1,755*
	Raut_3 [9]	NCBI under ID PRJNA416942	Sandy loam soils/ 380-500 ppm (treatment), 250-380 ppm (control)	V4-5	423,954 (17,664)	8,043	1,554*
	Xia [10]	Provided by the author(s)	All elevated CO_2 and control plots	V4-5	14,565 (2,427)	1,218	719**
	Yang [11]	NCBI under ID PRJNA422013	All elevated CO_2 and control plots	V4	53,227 (6,653)	4,025	1,463*
P addition	Leff_1 [12]	NCBI under ID PRJNA272747	frue.ch	V4	290,713 (32,301)	6,630	2,069*
	Leff_2 [12]	NCBI under ID PRJNA272747	mtca.au	V4	189,312 (21,034)	4,373	1,307*
	Leff_3 [12]	NCBI under ID PRJNA272747	ukul.za	V4	197,820 (21,980)	4,880	1,232*
	Leff_4 [12]	NCBI under ID PRJNA272747	summ.za	V4	147,895 (24,649)	4,079	1,530*
	Leff_5 [12]	NCBI under ID PRJNA272747	gilb.za	V4	248,399 (27,599)	5,196	1,545*
	Leff_6 [12]	NCBI under ID PRJNA272747	burrawan.au	V4	177,456 (19,717)	7,530	1,275*
Liming	Guo [13]	Provided by the author(s)	HC and control plots	V3–V4	34,489 (4,311)	1,643	945*

*OTUs present in half of plots or more

**OTUs present in 2 plots or more

Table S2. Mantel correlation coefficient of branch lengths between OTUs in neighbor-joining and maximum likelihood trees (all *P* < 0.001), and the significance (*P* values) of positive and negative responses of the consenTRAIT algorithm (testing whether the response is significantly associated with phylogeny) using maximum likelihood trees (compare to Table 2).

Perturbation	Locations	Mantel coefficient	Positive response*	Negative response*
Warming	Che [1]	**0.81**	**0.029**	**0.001**
	DeAngelis_1 [2]	**0.79**	**0.001**	**0.010**
	DeAngelis_2 [2]	**0.82**	0.754	**0.011**
	DeAngelis_3 [2]	**0.80**	0.125	**0.001**
	Waghmode [3]	**0.81**	**0.001**	**0.001**
	Zhang [4]	**0.85**	**0.002**	0.577
	Merging locations	**0.78**	**0.032**	**0.010**
Drought	Bastida_1 [5]	**0.74**	**0.015**	**0.003**
	Bastida_2 [5]	**0.83**	**0.010**	0.504
	Bouskill_1 [6]	**0.87**	**0.008**	0.486
	Bouskill_2	**0.86**	0.379	**0.008**
	Fernandes_1 [7]	**0.81**	0.417	**0.001**
	Fernandes_2 [7]	**0.78**	*0.056*	**0.001**
	Zhang [4]	**0.79**	0.180	0.366
	Merging locations	**0.82**	0.160	**0.028**
Elevated CO_2	Deng [8]	**0.80**	0.358	**0.003**
	Raut_1 [9]	**0.85**	0.640	0.210
	Raut_2 [9]	**0.86**	0.567	**0.001**
	Raut_3 [9]	**0.84**	**0.001**	0.384
	Xia [10]	**0.88**	0.195	0.574
	Yang [11]	**0.87**	0.482	**0.007**
	Merging locations	**0.82**	0.551	**0.050**
P addition	Leff_1 [12]	**0.89**	**0.044**	0.889
	Leff_2 [12]	**0.86**	0.104	**0.044**
	Leff_3 [12]	**0.85**	*0.077*	0.691
	Leff_4 [12]	**0.79**	0.501	0.144
	Leff_5 [12]	**0.84**	0.369	**0.001**
	Leff_6 [12]	**0.82**	0.103	*0.096*
	Merging locations	**0.82**	0.680	**0.008**
Liming	Guo [13]	**0.82**	**0.003**	**0.004**

*Italic and bold values indicate marginal (*P < 0.1*) and significant (*P < 0.05*) responses, respectively

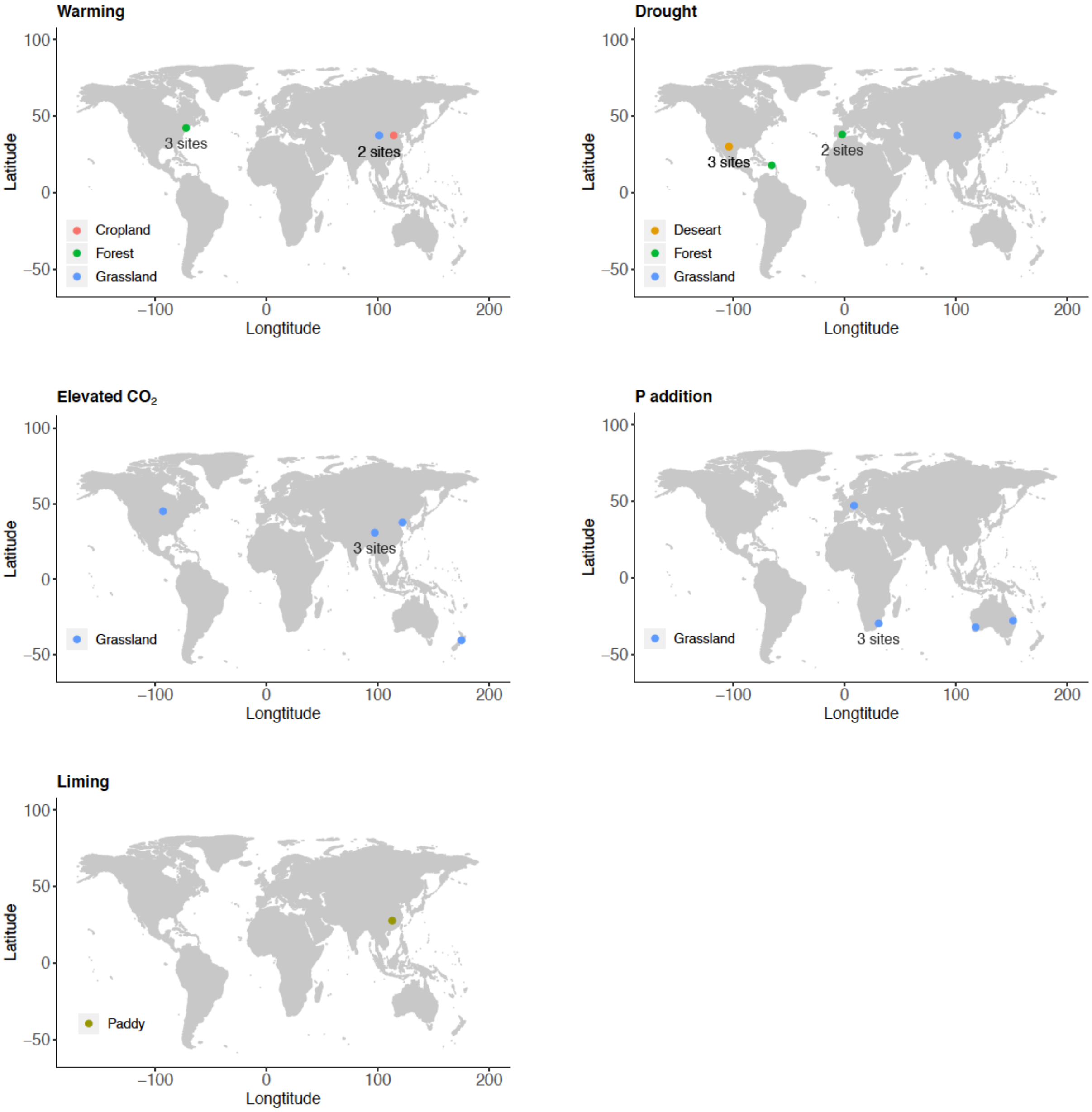

Figure S1. Locations of the global change experiments. Some symbols overlap because of their close proximity to one another.

Warming

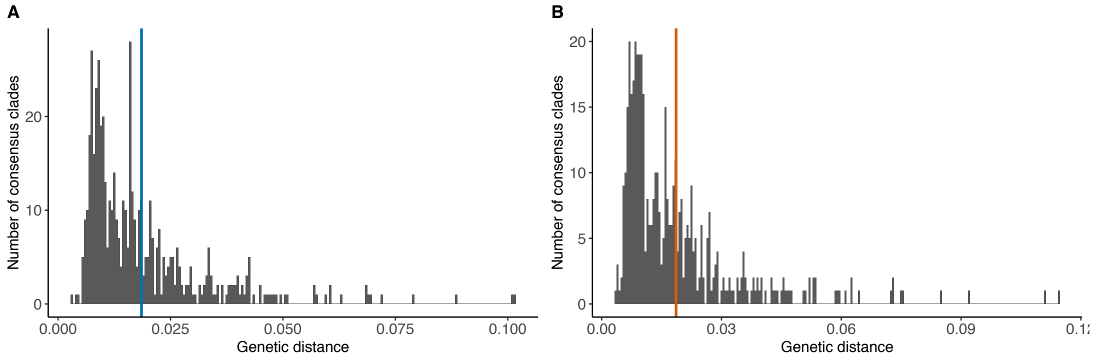

Drought

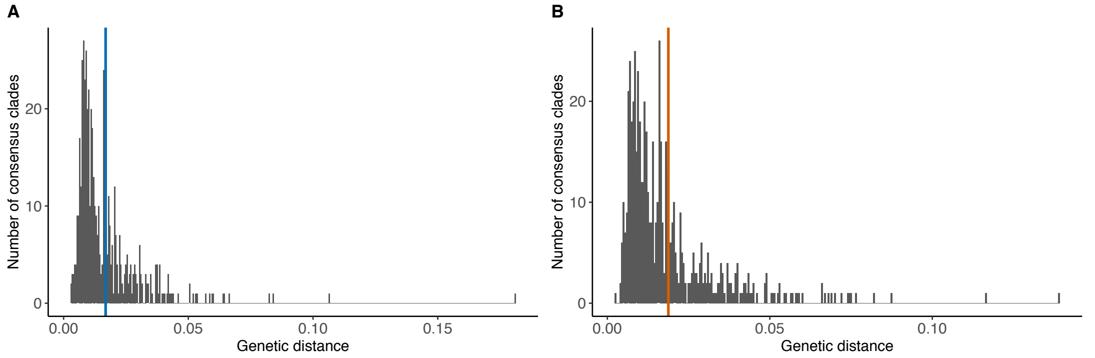

Elevated CO_2

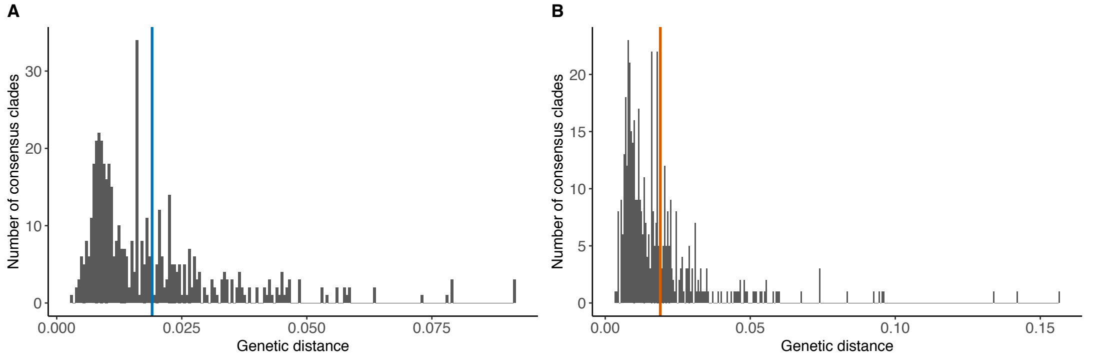

P addition

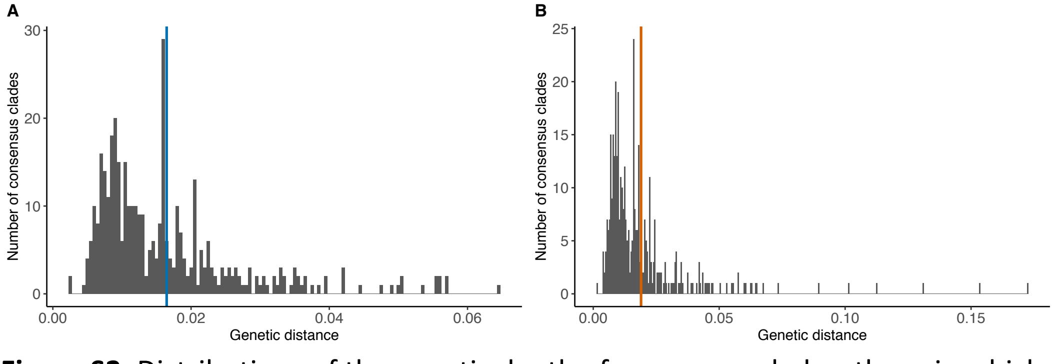

Figure S2. Distributions of the genetic depth of consensus clades, those in which >90% of the descendant OTUs show the same direction of response. For each perturbation, panel (A) shows the positive, and panel (B) negative, responses. The vertical blue or red line shows the mean genetic depth.

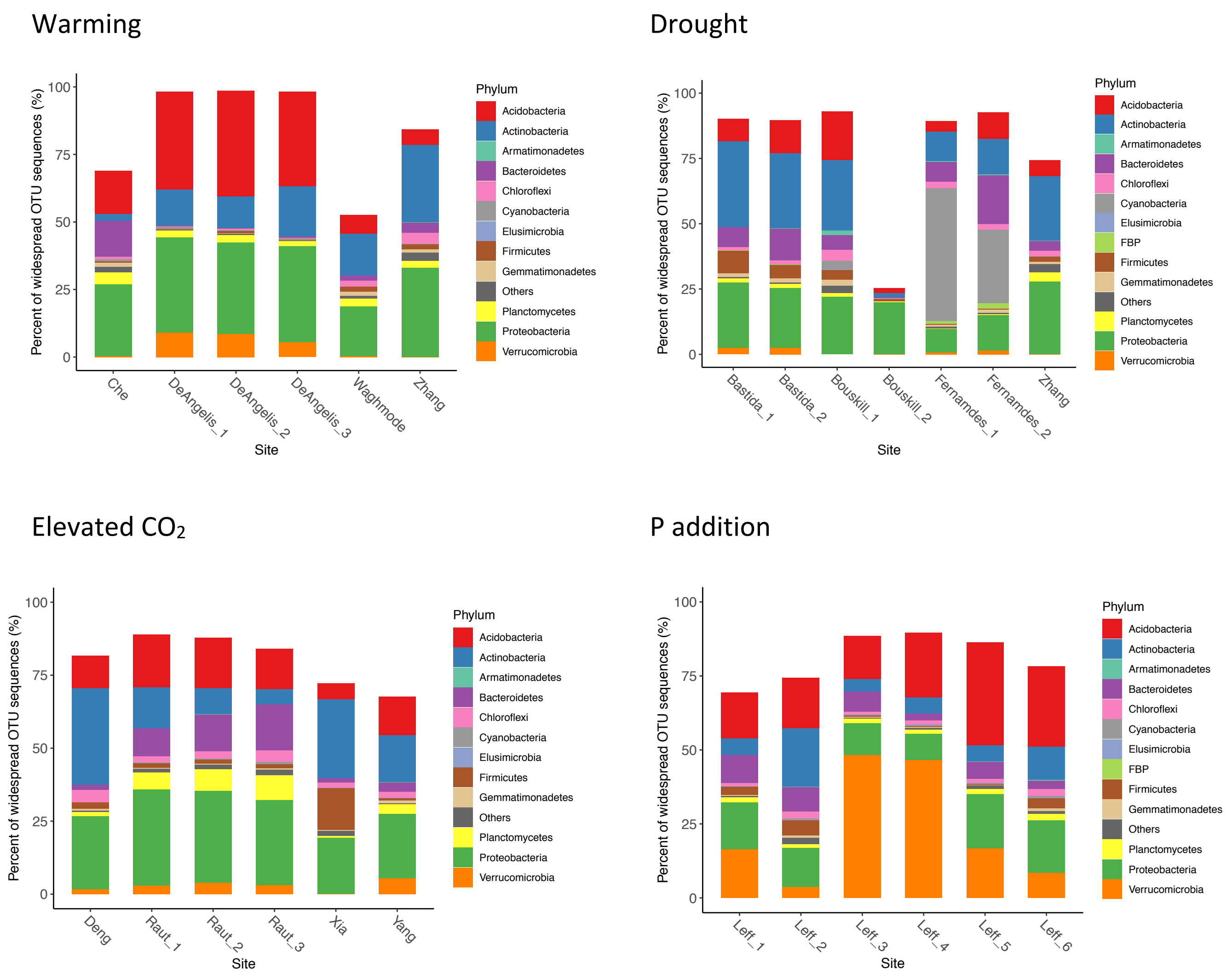

Figure S3. Percent of widespread OTUs (i.e., present >3 locations) out of the total number of sequences from each location. Colors indicate the phylum of the widespread OTUs.

References

1. Che R *et al.* 2018 Long-term warming rather than grazing significantly changed total and active soil procaryotic community structures. *Geoderma* **316**, 1–10. (doi:10.1016/j.geoderma.2017.12.005)
2. DeAngelis KM, Pold G, Topçuoglu BD, van Diepen LTA, Varney RM, Blanchard JL, Melillo J, Frey SD. 2015 Long-term forest soil warming alters microbial communities in temperate forest soils. *Front. Microbiol.* **6**, 1–13. (doi:10.3389/fmicb.2015.00104)
3. Waghmode TR, Chen S, Li J, Sun R, Liu B, Hu C. 2018 Response of Nitrifier and Denitrifier Abundance and Microbial Community Structure to Experimental Warming in an Agricultural Ecosystem. *Front. Microbiol.* **9**, 1–12. (doi:10.3389/fmicb.2018.00474)
4. Zhang K, Shi Y, Jing X, He J-S, Sun R, Yang Y, Shade A, Chu H. 2016 Effects of Short-Term Warming and Altered Precipitation on Soil Microbial Communities in Alpine Grassland of the Tibetan Plateau. *Front. Microbiol.* **7**. (doi:10.3389/fmicb.2016.01032)
5. Bastida F *et al.* 2017 Differential sensitivity of total and active soil microbial communities to drought and forest management. *Glob. Chang. Biol.* **23**, 4185–4203. (doi:10.1111/gcb.13790)
6. Bouskill NJ, Lim HC, Borglin S, Salve R, Wood TE, Silver WL, Brodie EL. 2013 Pre-exposure to drought increases the resistance of tropical forest soil bacterial communities to extended drought. *ISME J.* **7**, 384–394. (doi:10.1038/ismej.2012.113)
7. Fernandes VMC, Machado de Lima NM, Roush D, Rudgers J, Collins SL, Garcia-Pichel F. 2018 Exposure to predicted precipitation patterns decreases population size and alters community structure of cyanobacteria in biological soil crusts from the Chihuahuan Desert. *Environ. Microbiol.* **20**, 259–269. (doi:10.1111/1462-2920.13983)
8. Deng Y *et al.* 2012 Elevated carbon dioxide alters the structure of soil microbial communities. *Appl. Environ. Microbiol.* **78**, 2991–2995. (doi:10.1128/AEM.06924-11)
9. Raut S, Polley HW, Fay PA, Kang S. 2018 Bacterial community response to a preindustrial-to-future CO_2 gradient is limited and soil specific in Texas Prairie grassland. *Glob. Chang. Biol.* **24**, 5815–5827. (doi:10.1111/gcb.14453)
10. Xia W, Jia Z, Bowatte S, Newton PCD. 2017 Impact of elevated atmospheric CO_2 on soil bacteria community in a grazed pasture after 12-year enrichment. *Geoderma* **285**, 19–26. (doi:10.1016/j.geoderma.2016.09.015)
11. Yang S *et al.* 2019 Long-term elevated CO_2 shifts composition of soil microbial communities in a Californian annual grassland, reducing growth and N utilization potentials. *Sci. Total Environ.* **652**, 1474–1481. (doi:10.1016/j.scitotenv.2018.10.353)
12. Leff JW *et al.* 2015 Consistent responses of soil microbial communities to elevated nutrient inputs in grasslands across the globe. *Proc. Natl. Acad. Sci.* **112**, 10967–10972. (doi:10.1073/pnas.1508382112)
13. Guo A, Ding L, Tang Z, Zhao Z, Duan G. 2019 Microbial response to $CaCO_3$ application in an acid soil in southern China. *J. Environ. Sci. (China)* **79**, 321–329. (doi:10.1016/j.jes.2018.12.007)
14. Isobe K, Allison SD, Khalili B, Martiny AC, Martiny JBH. 2019 Phylogenetic conservation of bacterial responses to soil nitrogen addition across continents. *Nat. Commun.* **10**, 2499. (doi:10.1038/s41467-019-10390-y)

1. Che R *et al.* 2018 Long-term warming rather than grazing significantly changed total and active soil procaryotic community structures. *Geoderma* **316**, 1–10. (doi:10.1016/j.geoderma.2017.12.005)
2. DeAngelis KM, Pold G, Topçuoglu BD, van Diepen LTA, Varney RM, Blanchard JL, Melillo J, Frey SD. 2015 Long-term forest soil warming alters microbial communities in temperate forest soils. *Front. Microbiol.* **6**, 1–13. (doi:10.3389/fmicb.2015.00104)
3. Waghmode TR, Chen S, Li J, Sun R, Liu B, Hu C. 2018 Response of Nitrifier and Denitrifier Abundance and Microbial Community Structure to Experimental Warming in an Agricultural Ecosystem. *Front. Microbiol.* **9**, 1–12. (doi:10.3389/fmicb.2018.00474)
4. Zhang K, Shi Y, Jing X, He J-S, Sun R, Yang Y, Shade A, Chu H. 2016 Effects of Short-Term Warming and Altered Precipitation on Soil Microbial Communities in Alpine Grassland of the Tibetan Plateau. *Front. Microbiol.* **7**. (doi:10.3389/fmicb.2016.01032)
5. Bastida F *et al.* 2017 Differential sensitivity of total and active soil microbial communities to drought and forest management. *Glob. Chang. Biol.* **23**, 4185–4203. (doi:10.1111/gcb.13790)
6. Bouskill NJ, Lim HC, Borglin S, Salve R, Wood TE, Silver WL, Brodie EL. 2013 Pre-exposure to drought increases the resistance of tropical forest soil bacterial communities to extended drought. *ISME J.* **7**, 384–394. (doi:10.1038/ismej.2012.113)
7. Fernandes VMC, Machado de Lima NM, Roush D, Rudgers J, Collins SL, Garcia-Pichel F. 2018 Exposure to predicted precipitation patterns decreases population size and alters community structure of cyanobacteria in biological soil crusts from the Chihuahuan Desert. *Environ. Microbiol.* **20**, 259–269. (doi:10.1111/1462-2920.13983)
8. Deng Y *et al.* 2012 Elevated carbon dioxide alters the structure of soil microbial communities. *Appl. Environ. Microbiol.* **78**, 2991–2995. (doi:10.1128/AEM.06924-11)
9. Raut S, Polley HW, Fay PA, Kang S. 2018 Bacterial community response to a preindustrial-to-future CO_2 gradient is

limited and soil specific in Texas Prairie grassland. *Glob. Chang. Biol.* **24**, 5815–5827. (doi:10.1111/gcb.14453)

10. Xia W, Jia Z, Bowatte S, Newton PCD. 2017 Impact of elevated atmospheric CO2 on soil bacteria community in a grazed pasture after 12-year enrichment. *Geoderma* **285**, 19–26. (doi:10.1016/j.geoderma.2016.09.015)
11. Yang S *et al.* 2019 Long-term elevated CO 2 shifts composition of soil microbial communities in a Californian annual grassland, reducing growth and N utilization potentials. *Sci. Total Environ.* **652**, 1474–1481. (doi:10.1016/j.scitotenv.2018.10.353)
12. Leff JW *et al.* 2015 Consistent responses of soil microbial communities to elevated nutrient inputs in grasslands across the globe. *Proc. Natl. Acad. Sci.* **112**, 10967–10972. (doi:10.1073/pnas.1508382112)
13. Guo A, Ding L, Tang Z, Zhao Z, Duan G. 2019 Microbial response to CaCO 3 application in an acid soil in southern China. *J. Environ. Sci. (China)* **79**, 321–329. (doi:10.1016/j.jes.2018.12.007)
